# Comparative Genomics of *Leuconostoc carnosum*

**DOI:** 10.3389/fmicb.2020.605127

**Published:** 2021-01-11

**Authors:** Francesco Candeliere, Stefano Raimondi, Gloria Spampinato, Moon Yue Feng Tay, Alberto Amaretti, Joergen Schlundt, Maddalena Rossi

**Affiliations:** ^1^Department of Life Sciences, University of Modena and Reggio Emilia, Modena, Italy; ^2^Nanyang Technological University Food Technology Centre (NAFTEC), Singapore, Singapore; ^3^School of Chemical and Biomedical Engineering, Nanyang Technological University, Singapore, Singapore; ^4^Biogest-Siteia, University of Modena and Reggio Emilia, Modena, Italy

**Keywords:** *Leuconostoc carnosum*, genomics, pangenome analysis, bacteriocin, metabolism

## Abstract

*Leuconostoc carnosum* is a known colonizer of meat-related food matrices. It reaches remarkably high loads during the shelf life in packaged meat products and plays a role in spoilage, although preservative effects have been proposed for some strains. In this study, the draft genomes of 17 strains of *L. carnosum* (i.e., all the strains that have been sequenced so far) were compared to decipher their metabolic and functional potential and to determine their role in food transformations. Genome comparison and pathway reconstruction indicated that *L. carnosu*m is a compact group of closely related heterofermentative bacteria sharing most of the metabolic features. Adaptation to a nitrogen-rich environment, such as meat, is evidenced by 23 peptidase genes identified in the core genome and by the autotrophy for nitrogen compounds including several amino acids, vitamins, and cofactors. Genes encoding the decarboxylases yielding biogenic amines were not present. All the strains harbored 1–4 of 32 different plasmids, bearing functions associated to proteins hydrolysis, transport of amino acids and oligopeptides, exopolysaccharides, and various resistances (e.g., to environmental stresses, bacteriophages, and heavy metals). Functions associated to bacteriocin synthesis, secretion, and immunity were also found in plasmids. While genes for lactococcin were found in most plasmids, only three harbored the genes for leucocin B, a class IIa antilisterial bacteriocin. Determinants of antibiotic resistances were absent in both plasmids and chromosomes.

## Introduction

*Leuconostoc* is a genus of heterofermentative Lactobacillaceae (Zheng et al., 2020). The general traits of *Leuconostoc* species include facultative anaerobiosis, intrinsic vancomycin resistance, catalase negativity, ovococcoid morphology, and dextran production. At the time of writing, the genus *Leuconostoc* encompassed at least 20 recognized species or subspecies, mostly isolated from plant materials, dairy products, meats, and fermented vegetables (Jeon et al., 2017; Chen et al., 2020). Among them, *Leuconostoc carnosum* is a known colonizer of food matrices, particularly the meat-related ones (Shaw and Harding, 1989; van Laack et al., 1992; Parente et al., 1996; Budde et al., 2003; Geeraerts et al., 2018; Raimondi et al., 2018, 2019).

*Leuconostoc carnosum* is reported to grow during the first days of the shelf-life in packaged meat products, which may range up to some weeks. The charge of contaminant bacteria, often dominated by *L. carnosum*, tends to increase and peaks up to 10^8^ cells/g in some foods (Raimondi et al., 2018, 2019). These remarkably high levels of *L. carnosum* contamination were observed in modified atmosphere packaged (MAP) cooked ham, where it prevailed over the microbiota well before the expiry date (Björkroth et al., 1998; Vasilopoulos et al., 2010; Raimondi et al., 2019); in other meat products, such as sausages, vacuum-packaged smoked bacon, and sliced cooked poultry (Geeraerts et al., 2018; Li et al., 2019); and in processed vegetables (Hong et al., 2014; Jung et al., 2014). As a result, a high load of living *L. carnosum* may be ingested with certain foods, including many popular ready-to-eat products for which the consumption is steadily increasing all over in the world. Nonetheless, any potential impact of *L. carnosum* on human health has never been assessed.

The high moisture and the low salt content, the near-neutral pH, and the availability of nutrients of meat-related products encourage microbial growth, eventually leading to spoilage. With respect to food wholesomeness, the role of *L. carnosum* is controversial. Many studies describe it as a spoilage bacterium that causes meat deterioration and affects sensorial properties by souring, discoloration, gas production, and slime formation (Shaw and Harding, 1989; Björkroth et al., 1998; Samelis et al., 2006; Raimondi et al., 2019). On the other hand, likewise other *Leuconostoc* species, *L. carnosum* yields organic acid and hydrogen peroxide, which exert an intrinsic antimicrobial effect, and can produce class II heat-stable bacteriocins (van Laack et al., 1992; Felix et al., 1994; Hastings et al., 1994; Stiles, 1994). Typical bacteriocins produced by *L. carnosum* are leucocins, consisting of small peptides of 30–50 residues with differences due to the length of the prepeptide sequence. Their genetic information generally resides in plasmids but can occur also in the chromosome. Most of leucocins are active against both *Listeria monocytogenes* and *Listeria innocua*, but can also inhibit some lactic acid bacteria, especially at low pH (Felix et al., 1994; Parente et al., 1996; Wan et al., 2015).

Contamination of meat products with *L. carnosum* may restrict the growth of spoiling and pathogenic bacteria (Jacobsen et al., 2003; Lawton et al., 2020). As a matter of fact, the capability of *L. carnosum* to deteriorate meat products seems to be a strain-specific feature. A survey of MAP cooked ham samples from different producers revealed that, at the end of the shelf life, *L. carnosum* dominated the microbiota of both the good and the spoiled ones (Raimondi et al., 2019). These evidences opened the perspective to develop protective starters with specifically selected strains of *L. carnosum* that may preserve meat without impacting on the sensorial properties (Budde et al., 2003).

The dual role of *L. carnosum* in spoilage and preservation, the high loads of these bacteria entering the human diet and possibly affecting health, and the lack of studies aimed to highlight strain to strain differences prompted us to explore more deeply in this species. In the present study, comparative genomics was performed to explore the main genetic determinants of all the 17 strains of *L. carnosum* that have been sequenced so far. Twelve strains were recently isolated from meat matrices and newly sequenced (Candeliere et al., 2020), whereas five had a genome already publicly available (Table 1; Jung et al., 2012). Using the dataset of the 17 chromosomal sequences, a comparative genome analysis of the *L. carnosum* taxon was undertaken through the assessment of the phylogeny and of pan- and core genome. Genetic diversity, plasmids, phages, Clustered Regularly Interspaced Short Palindromic Repeats (CRISPR-Cas) systems, bacteriocin, antibiotic resistances, and metabolic capabilities were also investigated.

**TABLE 1:** General genomic features of the 17 strains of *Leuconostoc carnosum* analyzed in this work.

Strains	Genome size (bp)	No. of contigs	N50	L50	Coverage	G + C (%)	No. of CDSs	No. of tRNA	No. of pseudogenes	No. of prophages	Plasmids
WC0318	1,745,630	15	1142374	1	638	37.2	1,739	51	55	2	pFRA18 and pALB18
WC0319	1,700,071	11	1137580	1	521	37.1	1,679	49	41	1	pELI19
WC0320	1,812,114	18	1109738	1	561	37.1	1,853	51	58	3	pFRA20, pALB20, and pICCOLO
WC0321	1,804,293	40	1106035	1	532	37.1	1,854	51	54	2	pFRA21, pGLO21, and pALB21
WC0322	1,853,239	16	306690	2	515	37.0	1,898	50	50	3	pALB22 and pLQ22
WC0323	1,773,698	23	256026	3	560	37.2	1,802	51	49	2	pFRA23 and pGLO23
WC0324	1,765,760	13	1137276	1	460	37.1	1,768	49	39	3	pELI24
WC0325	1,830,248	27	229291	3	502	37.2	1,881	51	59	4	pLQ25 and Piccolo
WC0326	1,770,048	22	412621	2	574	37.2	1,767	49	54	1	pSTE
WC0327	1,769,683	20	412621	2	568	37.2	1,768	49	53	1	pSTE
WC0328	1,815,949	36	239739	3	538	37.1	1,826	51	51	3	pALAN28, pUFO, and pELI28
WC0329	1,650,966	14	256123	3	455	37.2	1,644	51	49	1	pFRO29
JB16	1,645,096	5	1645096	1	–	37.2	1,686	64	30	1	pKLC1, pKLC2, pKLC3, and pKLC4
CBA3620	1,590,008	3	1590008	1	–	37.4	1,635	66	31	3	unnamed1 and unnamed2
MFPC16A2803	1,786,865	50	242386	3	–	37.0	1,846	32	64	2	pMFPC16A2803B and pMFPC16A28E
MFPA29A1405	1,634,774	22	165377	4	–	37.3	1,660	46	40	2	pMFPA29A1405B and pMFPA2A1405C
DSM 5576T	1,820,660	21	147749	4	–	37.0	1,740	47	–	1	Not yet identified

## Materials and Methods

### Genomes and Genomes Analysis

The 12 genomes of *L. carnosum* strains published by Candeliere et al. (2020) are available with the following GenBank accessions: SAMN11618753, SAMN11618754, SAMN11618755, SAMN11 618756, SAMN11618757, SAMN11618758, SAMN11618759, SAMN11618761, SAMN11618763, SAMN11618764, SAMN11618767, and SAMN11618768. The genomes of the other *L. carnosum* strains, i.e., JB16, CBA3620, MFPC16A2803, MFPA29A1405, and DSM 5576T, are available with the accessions SAMN02603179, SAMN11843679, SAMEA104699786, SAMEA104699785, and SAMN14908560, respectively. The strains were isolated from different sources, mostly meat-related products: MAP cooked ham (WC0318, WC0319, WC0320, WC0321, WC0322, WC0323, WC0324, WC0325, and WC0329), MAP sausages (WC0326, WC0327, and WC0328), vacuum-packed beef carpaccio (MFPC16A2803), MAP beef carpaccio (MFPA29A1405), vacuum-packaged beef (DSM 5576T), and kimchi (JB16 and CB3620).

Plasmids contigs were identified using plasmidSPAdes v 3.12 (Nurk et al., 2017). Average Nucleotide Identity (ANI) and digital-DNA/DNA hybridization (dDDH) were calculated utilizing the web tools ANI Matrix^1^ and Genome-to-Genome Distance Calculator GGDC 2.1^2^, by all-against-all approach (Meier-Kolthoff et al., 2013; Rodriguez-R and Konstantinidis, 2016). The thresholds for species demarcation were 95 and 70% for ANI and dDDH, respectively (Richter and Rosselló-Móra, 2009).

Gene clusters, such as prophages, CRISPR/Cas systems, insertion sequences (ISs), bacteriocins genes, and antimicrobial resistances, were identified with specific web tools. Prophage sequences were searched with PHASTER^3^ (Arndt et al., 2016). CRISPRs and Cas genes were searched utilizing CRISPRCasFinder^4^ (Couvin et al., 2018), with default settings and subtype clustering of Cas genes. Bacteriocins genes were searched with BAGEL 4 server^5^ (van Heel et al., 2018). ISs were identified with ISfinder^6^) (Siguier et al., 2006). Antimicrobial resistance was assessed by Resistance Gene Identifier (RGI) tool of Comprehensive Antibiotic Resistance Database (CARD), processing the contigs file for “Perfect, Strict, and Loose hits^7^” (Alcock et al., 2020).

### Genomes Annotation and Functional Characterization

Genomes were annotated with Prokka, utilizing default parameters (Seemann, 2014). Roary was utilized to calculate the pangenome and to delineate core, soft core, accessory, shell, and cloud genes, utilizing Prokka annotation files (Page et al., 2015). To differentiate plasmids and chromosomal accessory genes and highlight the plasmid contribution, Roary was used to calculate the pangenome with and without the plasmid contigs. COG annotation was conducted utilizing WebMGA server^8^ (Wu et al., 2011), using as input the protein files predicted by Prokka. A phylogenetic tree based on core genome alignment was constructed with FastTree (Price et al., 2009) utilizing the alignment file produced by Roary and was visualized with iTOL (Letunic and Bork, 2019).

To investigate the similarity among genomes and among plasmids, Jaccard’s distance was computed based on the presence/absence of predicted genes and subjected to Principal Coordinate Analysis (PCoA).

The functional prediction of the genomes was carried out with the KEGG tools BlastKOALA^9^ and Mapper^10^ with the aim to predict the metabolic functions, such as transporters, sugars catabolism, and biosynthetic pathways of amino acids, vitamins, and bases (Kanehisa et al., 2016; Kanehisa and Sato, 2020).

The presence of specific genes that are not included in KEGG annotation was investigated with a BLASTp search of cognate proteins recognized in a closely related taxon (i.e., in *L. carnosum*, in the genus *Leuconostoc*, in other Lactobacillales, or in other bacteria). The following enzymes for citrate metabolism and acetoin pathway were searched (García-Quintáns et al., 2008): citrate permease (AAA60396) of *Leuconostoc mesenteroides* subsp. *mesenteroides*, citrate lyase alpha and beta subunits (CAA71633 and CAA71632) of *L. mesenteroides* subsp. *cremoris*, oxaloacetate decarboxylase (AFS39629) of *Leuconostoc gelidum*, alpha-acetolactate synthase (SPJ44178) of *L. carnosum*, alpha-acetolactate decarboxylase (AFT82058) of *L. carnosum*, diacetyl reductase (SPJ42929) of *L. carnosum*, and 2,3-butanediol dehydrogenase (WP_135197409) of *L. carnosum*. The genes encoding the carboxylases yielding biogenic amines were searched (Li et al., 2018; Rodrigo-Torres et al., 2019): tyrosine decarboxylase (AAN77279) and agmatine deaminase (ABS19476 and ABS19477) of *Lactobacillus brevis* (proposed *Levilactobacillus brevis*), ornithine decarboxylase (ANJ65946) of *Lactobacillus rossiae* (proposed *Furfurilactobacillus rossiae*), histidine decarboxylase (N877767) of *Lactobacillus reuteri* (proposed *Limosilactobacillus reuteri*), and lysine decarboxylase (NP_414728) of *Escherichia coli*. For menaquinone biosynthesis, the presence of 1,4-dihydroxy-2-naphthoyl-CoA hydrolase from *L. mesenteroides* subsp. *mesenteroides* ATCC 8293 (ABJ61187) was searched.

## Results

### General Genome Features

The 12 newly assembled genomes of *L. carnosum* had a mean size of 1.77 Mbp, laying in the range between 1.65 and 1.85 Mbp of strains *L. carnosum* WC0329 and *L. carnosum* WC0322, respectively, and a GC content of 37.0–37.2% (Table 1). The genomes of the five already published strains had a size of 1.59–1.79 Mbp and a GC content of 37.0–37.4% (Table 1). A > 455-fold coverage was obtained for all the 12 genomes, with a mean of 535.3-fold. The draft genomes encompassed from 11 to 40 contigs, on average 21 per genome. For all the assemblies, 1–3 contigs together constituted more than 50% of the genome size (L50 value, mean = 1.92; N50 value, mean = 645,510 bp) (Table 1). A total of 20 natural plasmids, ranging in size from 2.4 to 63.5 kbp, were identified in the new 12 draft genomes, 1–3 per strain (Tables 1, 2). Twelve other plasmids occurred in four already available genomes (Table 2), whereas the information was not available for *L. carnosum* DSM 5576T. For all the pairwise comparisons between 17 genomes, both ANI and dDDH were higher than the corresponding threshold for species demarcation (95 and 70%, respectively); thus, all the strains were confirmed to belong to the same species with only minor intra-species differences (Supplementary Table 1). *L. carnosum* WC326 and WC327 resulted in the same strain according to both ANI and dDDH.

**TABLE 2:** Size and number of CDSs predicted in the plasmids.

Plasmid	Size (bp)	No. of CDSs
pALAN28	63,454	72
pALB18	27,911	32
pALB20	42,988	45
pALB21	29,994	37
pALB22	41,757	49
pELI19	18,422	21
pELI24	13,067	14
pELI28	9,860	12
pFRA18	32,446	34
pFRA20	63,088	67
pFRA21	42,529	47
pFRA23	48,616	51
pFRO29	50,548	57
pGLO21	41,385	40
pGLO23	39,823	41
pICCOLO	2,351	3
pLQ22	36,356	44
pLQ25	42,858	41
pSTE	39,016	37
pUFO28	18,346	16
pKLC1	21,990	21
pKLC2	29,615	34
pKLC3	40,165	45
pKLC4	36,602	39
unnamed1	57,926	69
unnamed2	53,399	67
pMFPC16A2803B	22,807	36
pMFPC16A28E	2,344	3
pMFPA29A1405B	18,286	24
pMFPA2A1405C	13,778	14
pMFPC16A2803A	78,165	88
pMFPC16A2803C	11,421	12

**FIGURE 1:**
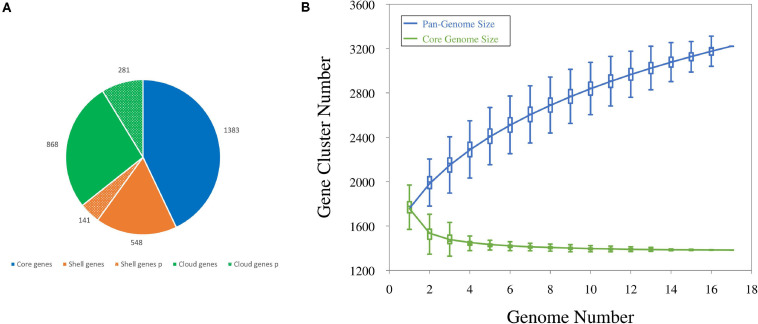
**(A)** Distributions of CDSs found in the pangenome of *L. carnosum*: core genes (blue), shell genes (orange), and cloud genes (green) in chromosome (plain colors) and plasmids (dotted colors). **(B)** Estimation of the pangenome (blue) and the core genome (green) by including genomes one by one.

The number of coding sequences (CDSs) in the 12 new genomes ranged between 1,644 and 1,898, with a mean of 1,790. Single plasmids harbored from 3 to 67 genes; thus, each draft genome included 14–124 plasmid genes, accounting for 0.8–6.7% of the CDS content. The number of CDSs in the five genomes already available ranged from 1,635 and 1,846, with plasmids of four strains harboring from 3 to 69 genes. All the CDSs in the 17 genomes were compared by a blast all-against-all approach to identify orthologous gene groups and construct pan- and core genome matrices. The pangenome of *L. carnosum* encompassed 3,221 orthologous genes, whereas the core genome included 1,383 chromosomal genes (on average 43% of the total genes). The accessory genome was composed of 689 shell genes (548 of which in the chromosome and 141 in plasmids) and 1,149 cloud genes (868 of which in the chromosome and 281 in plasmids) (Figure 1A). As per the Heap’s law, the value of γ was 0.35 for 17 genomes, and thus *L. carnosum* pangenome was considered open (Figure 1B; Tettelin et al., 2008).

**FIGURE 2:**
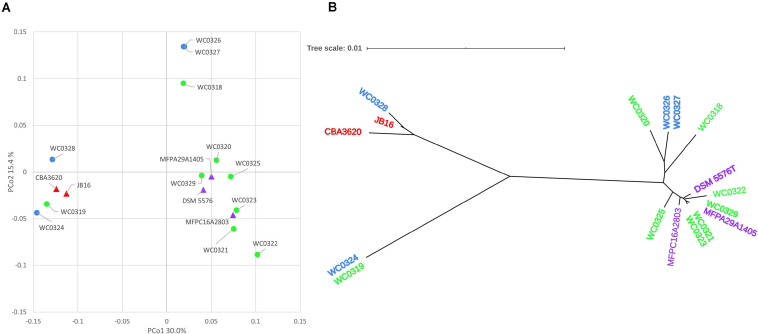
**(A)** PCoA visualization of Jaccard distances among genomes, based on shared genes. Circles are the genomes published by Candeliere et al. (2020); triangles are the genomes already published. **(B)** Neighbor joining unrooted phylogenetic tree based on core genome alignment. In both panels, colors indicate the source of the strains: MAP cooked ham, green; MAP sausage, blue; packaged beef products, purple; and kimchi, red.

The Jaccard distance matrix among genomes, due to the presence/absence of the genes, is displayed in the PCoA plot of Figure 2A. Five genomes laying at negative values of PCo1 (i.e., WC0319, WC0324, WC0328, JB16, and CBA3620) were separated from the others dispersed at positive values of PCoA1, among which three lay at positive values of PCo2 (WC0318, WC0326, and WC0327), and the others grouped together at lower values. The phylogenetic relationship between the 17 strains was constructed using relative hierarchical clustering based on core genome alignment (Figure 2B). The low phylogenetic distances confirmed a close relatedness among the *L. carnosum* strains, even though sequence differences within the core genome revealed three major clades. All the genomes that lay at positive PCo1 values were in the same phylogenetic clade, whereas the five genomes that lay at negative PCo1 values were clearly separated in two different clades.

**FIGURE 3:**
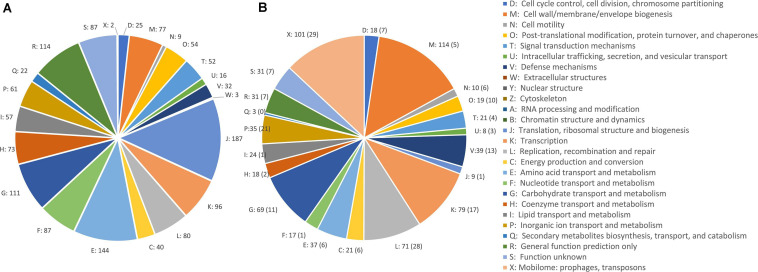
COG class abundances in core genome **(A)** and in the accessory genome **(B)**. The number of CDSs predicted within each class is reported. The CDSs in plasmids are in brackets.

A comparative analysis of chromosomal sequences was carried out for all the genomes. The 1,383 genes of the core genome were ascribed to 24 COG families (Figure 3). Functional distribution of the COGs of the core showed that the majority encoded components of the information processing systems associated to translation, ribosomal structure, and biogenesis (13.1%) and of amino acid transport and metabolism (10.1%). The core also included 87 genes of function unknown (6.1%) and 114 genes with only a general prediction of biochemical activity (8.0%).

### Mobilome

The chromosome sequences were analyzed for the presence of mobile elements, such as genes putatively encoding ISs and transposases. Twenty-nine genes encoding transposases were identified in the accessory genome, 20 of them as part of IS. Except for *L. carnosum* MFPA29A1405 that did not harbor any, all the strains harbored at least one IS. The ISs belonged to the families IS3, IS5, IS6, IS30, IS256, and IS1182. The IS presented similarities with counterparts of other Lactobacillales: IS3, *Lactobacillus sakei* (proposed *Latilactobacillus sakei* by Zheng et al., 2020) and *Weissella cibaria*; IS5 and IS1182, *Lactobacillus plantarum* (proposed *Lactiplantibacillus plantarum* by Zheng et al., 2020); IS6, *Enterococcus faecium* and *L. mesenteroides*; IS30, *L. plantarum*, *Leuconostoc lactis*, and *Pediococcus pentosaceus*; and IS256, *Enterococcus hirae* and *Lactobacillus helveticus*. The most represented were IS3 and IS30 transposases (seven and six genes, respectively).

### Plasmids

**FIGURE 4:**
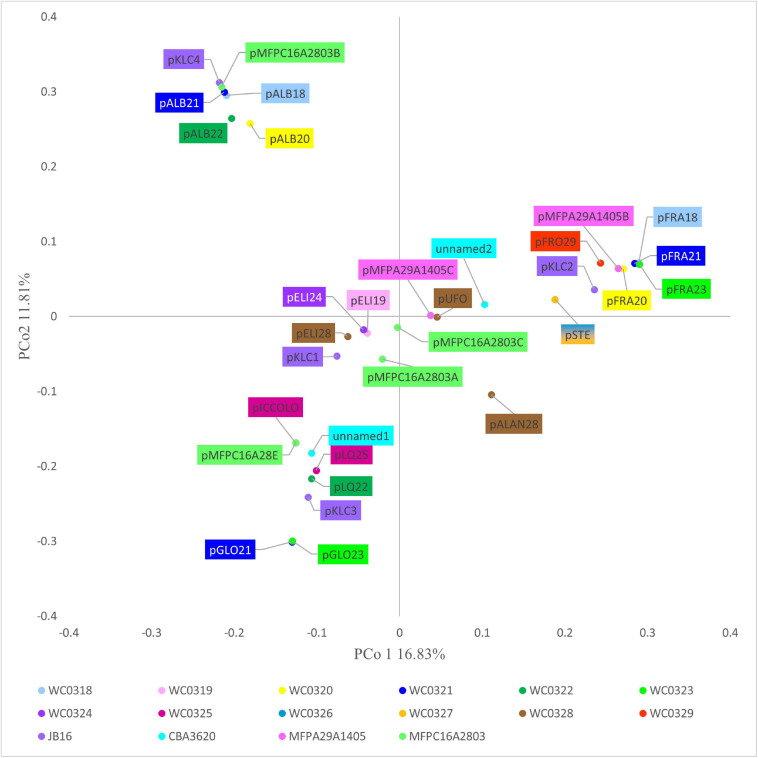
PCoA visualization of Jaccard distances among plasmids, based on shared genes.

The 32 plasmids were annotated (Supplementary Datasheet 1). The Jaccard distance matrix among plasmids, due to the presence of shared genes and predicted functions, is displayed in the PCoA plot of Figure 4.

pALB plasmids, pKLC4, and pMFPC16A2803B harbored genes encoding all the functions for conjugative transfer, including mating pair formation and DNA replication, mobilization, and transfer (Supplementary Datasheet 1). The series pGLO and pLQ and the plasmids pMFPA29A1405B, pMFPC16A28E, and pKLC1 had some conjugation genes, but lacked the genes encoding mobilization proteins (relaxases), resulting not mobilizable. The series pELI and pSTE, pFRO29, pALAN28, pUFO, pKLC2, pKLC3, pMFPC16A2803A, pMFPC16A2803C, pMFPA2A1405C, unnamed1, and unnamed2 were mobilizable but not conjugative plasmids, for the presence of 2–4 mobility-related genes.

pICCOLO and pMFPC16A28E were likely the same small plasmid of approximately predicted 2,350 bp, occurring in the strains *L. carnosum* WC0320, WC0325, and MFPC16A2803. They showed a very high identity (>97.8%) with the small plasmid pM411 (2,303 bp) of *L. plantarum*, replicating *via* the rolling circle mechanism. They encoded an *N*-acetyltransferase and the rolling circle initiator protein RepB. Genes encoding RepB proteins occurred in 16 of the 32 replicons, such as in the pELI series (Supplementary Datasheet 1). IS3 transposase was found in the series pFRA and in pSTE, pFRO29, pKLC2, pKLC3, pKLC4, pMFPC16A2803A, pMFPC16A2803C, pMFPA29A1405B, unnamed1, and unnamed2. pSTE and pKLC2 also shared IS30 transposase, whereas pFRO29, pMFPA29A1405B, and the series pFRA shared IS110 that contributed to their clustering. The series of pGLO plasmids harbored the IS6 transposase and other common recombinases and transposases, whereas the pSTE plasmids and pKLC2 encoded both IS3 and IS30 transposases. Peculiar was the scarce presence or absence of genes associated to transposases in pALB plasmids, albeit the large size of these replicons, likely due to their conjugative feature. The plasmid unnamed2 and series pALB and pELI were also characterized by genes encoding DNA-methyl transferase and/or restriction endonucleases.

Known antibiotic resistance genes were not identified in the new plasmids. Genes encoding ImmA/IrrE and RelE/ParE toxin–antitoxin systems were present in the series pSTE, pLQ25, and pGLO and in pKLC3, pMFPC16A2803A, and unnamed1. The latter also harbored RelB/DinJ system. ImmA/IrrE, RelE/ParE, and RelB/DinJ thus added to HicB family and PemK/MazF toxin–antitoxin systems located in the core genome. The plasmids often harbored genes involved in stress responses and participating in arsenic and heavy metal detoxification. Genetic determinants of thioredoxin were identified in pALB and pGLO series and in pKLC1, pKLC3, pKLC4, pMFPC16A2803A, pMFPC16A2803B, unnamed1, and unnamed2 plasmids. Interestingly, only pKLC1, pMFPC16A2803A, and pGLO series also encoded a glutathione reductase gene, the product of which can contribute for regulating cellular redox homeostasis, in concert with the glutathione peroxidase present in the core genome.

Genes associated to bacteriocin production were detected in four plasmids. pFRA21, pFRA23, and pFRO29 held the genes encoding the leucocin B and an immunity protein. Moreover, the genes encoding an additional immunity protein and an accessory secretion protein for the bacteriocin were predicted in pFRA21 and pFRA23. Conversely, pFRA18 did not harbor any gene associated to bacteriocin production. pALAN28 had three other genes linked to antibacterial activity: a lactococcin, a mesentericin, and another immunity protein.

### Reconstructed Metabolic Pathways

**FIGURE 5:**
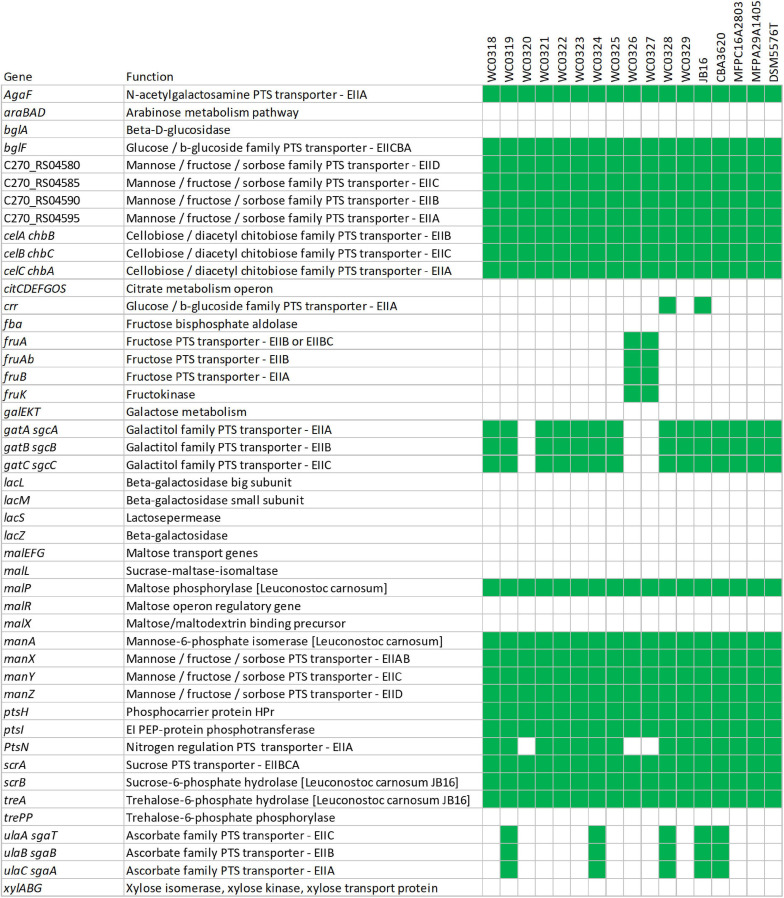
Genetic potential for metabolism of carbohydrates based on the presence (green) or absence (white) of predicted transporters or enzymes.

Pathway reconstruction based on KEGG gene annotation of the core genome predicted at least five complete phosphotransferase system (PTS) transporters, i.e., one for sucrose, two belonging to mannose–fructose–sorbose family, and one each to glucose–β-glucoside and cellobiose–diacetyl chitobiose families. On the other hand, a complete PTS transporter for fructose and two belonging to ascorbate and galactitol families were accessory. Genes encoding other PTS components were also recognized as part of incomplete transporters in the core or in the accessory genome, such as EIIA components (or proteins with EIIA domains) with similarity to *agaF*, *crr*, and *ptsN* that may play a role in the uptake of substrates if they could interact with unidentified EIIB and EIIC components (Figure 5 and Supplementary Datasheet 2).

Complete ABC transporters for the uptake of ribose and xylose, oligopeptides, many amino acids, and other compounds or nutrients, such as nucleosides, amines, biotin, phosphate, and manganese, were predicted in the core genome (Supplementary Datasheet 2). The accessory genome included a complete ABC transporter for the uptake of choline and two exporters presenting similarity to those encoded by the operons *TagGH* and *NodIJ* for the export of teichoic acid and capsular polysaccharides, respectively. Three eukaryotic-type ABC exporters were also recognized in the core genome. One is involved in cytochrome export and assembly, whereas two have similarity to the multidrug efflux systems *mdlAB* and *lmrCD*. The accessory genome included a further eukaryotic-type ABC transporter, similar to *blpA* that is involved in bacteriocin export (Supplementary Datasheet 2).

*Leuconostoc carnosum* is heterofermentative, with the sugars being metabolized *via* the pentose phosphate and phosphoketolase pathway (PKP), yielding lactate, acetate, or ethanol and CO_2_ (Supplementary Datasheet 3). The pivot enzyme of PKP, D-xylulose-5-phosphate phosphoketolase (EC 4.1.2.9), was encoded in the core genome. Consistently, the main bacterial glycolytic pathways were incomplete, the Embden–Meyerhof’s one lacking 6-phosphofructokinase and fructose-bisphosphate aldolase, and the Entner–Doudoroff’s one lacking phosphogluconate dehydratase and 2-dehydro-3-deoxy-phosphogluconate aldolase. Leloir’s pathway for galactose utilization was incomplete in all the strains, even though some strains harbored the genes encoding galactose epimerase (EC 5.1.3.3) and UDP-glucose 4-epimerase (EC 5.1.3.2). The whole route for ascorbate transformation into xylulose 5-phosphate and channeling into the pentose phosphate pathway was accessory, the seven enzymes being encoded in some strains, or all missing in the others (Supplementary Datasheet 3). The citrate pathway was absent in all the strains, due to the lack of the *Cit* genes encoding citrate transporter, citrate lyase, and oxaloacetate decarboxylase. Nonetheless, all the strains harbored the genes encoding α-acetolactate synthase, α-acetolactate decarboxylases, and diacetyl acetoin reductases and may yield acetoin and 2,3-butanediol from pyruvate.

A total of 25 peptidase genes were identified (Supplementary Figure 1). The vast majority was encoded by core genes, whereas a minority were accessory. Reconstruction of amino acid biosynthesis pathways suggested that the strains did not differ regarding the ability to produce amino acids, except for tryptophan (Supplementary Datasheet 3). Some metabolic routes involved in the addition of an amino group to carbon backbones seemed interrupted, thus hampering the synthesis of some amino acids. In particular, the genes encoding glutamate dehydrogenase and glutamate synthase for the biosynthesis of glutamate from 2-oxoglutarate were not identified. The lack of alanine dehydrogenase and aspartate aminotransferase interrupted the synthesis of alanine and aspartate, respectively, whereas the pathway of serine production from glycolysis intermediates seemed ineffective due to the lack of phosphoserine phosphatase.

The pathways depending on pre-formed serine to yield glycine and cysteine, on glutamate to yield glutamine, arginine, and proline, and on aspartate to yield asparagine, threonine, and methionine were complete in the core genome (Supplementary Datasheet 3). Many genes encoding enzymes of lysine biosynthesis from aspartate were predicted in the core genome. The biosynthetic pathway seemed complete from aspartate to 2,3,4,5-tetrahydrodipicolinate. Some enzymes ascribable to the bacterial routes transforming this intermediate into lysine (i.e., the succinylase, acetylase, and dehydrogenase pathways) could be predicted, yet none of the routes seemed complete due to the lack of one or two enzymes. Interestingly, the carboxy-lyase catalyzing lysine formation from the last intermediate onto which the three routes converge (meso-2,6-diaminoheptanedioate) was predicted.

The anabolic pathways leading to branched chain amino acids were all complete in the core genome. With regard to aromatic amino acids, the route from 5-phosphoribosyl diphosphate to histidine and the shikimate pathway from erythrose 4-phosphate to chorismate were complete. The pathway leading to tyrosine seemed interrupted due to the lack of chorismate mutase, whereas the pathway leading to phenylalanine also lacked prephenate dehydratase. Nonetheless, the other enzymes necessary for the synthesis of tyrosine and phenylalanine were predicted. The metabolic pathway for tryptophan was complete in most of the strains, whereas it was absent in *L. carnosum* WC0318, WC0319, and WC0324.

All the strains harbored the whole set of genes and operons for the synthesis of adenine and guanine ribonucleotides from inosine monophosphate and of pyrimidine ribonucleotides from UMP. On the other hand, the pathway for uridine monophosphate biosynthesis from glutamine missed the gene encoding the aspartate carbamoyltransferase regulatory subunit, whereas the catalytic subunit was present. For pyrimidine deoxyribonucleotide biosynthesis from CDP/CTP, all the strains lacked the gene encoding the dCTP deaminase.

All the strains seemed unable to synthetize any biogenic amine, based on the absence of the genes encoding histidine, lysine, tyrosine, and ornithine decarboxylases (*hdcA*, *ldc*, *tyrDC*, and *odc*, respectively) and agmatine deiminase (*aguD* and *aguA*).

The metabolic pathways for the biosynthesis of most vitamins and cofactors were missing or largely incomplete in all the strains that seemed unable to synthetize thiamine, riboflavin, pyridoxal, NAD, pantothenate, biotin, folate, heme, cobalamin, and ubiquinone. Only the pathway for menaquinone biosynthesis was complete in all the strains. In this pathway, the module encoding 1,4-dihydroxy-2-naphthoyl-CoA hydrolase, a thioesterase of PaaI family, was not predicted using KEGG but was found utilizing the sequence of the cognate protein of *L. mesenteroides* ATCC 8293 as query for a BLASTp search against the proteins of *L. carnosum*. A PaaI family thioesterase presenting 68% of identity was detected in all the strains, suggesting that they all have the whole set of genes for the biosynthesis of menaquinone.

### Stress Responses and Two-Component Signal Transduction Systems

The pangenome of *L. carnosum* lacked genetic determinants associated to superoxide dismutase, catalase, and *de novo* biosynthesis of glutathione. On the other hand, the core genome harbored other genes for coping with oxidative stress and reactive oxygen species, such as those encoding thioredoxin and thioredoxin reductase, a peroxidase, and two peroxiredoxins. Moreover, genes encoding thioredoxin-related proteins were harbored by 14 out of 32 plasmids. The core genome harbored the gene encoding a glutathione peroxidase (EC 1.11.1.9) that relies on pre-formed glutathione for H_2_O_2_ detoxification, whereas two plasmids encoded the counterpart encoding the glutathione reductase.

The genes of the stringent response mediated by the alarmone nucleotides (P)PPGPP were present in the core genome. Putative members of the two-component system were identified: six histidine kinases (HKs) and seven response regulators (RRs) were located in the core genome, and single HK and RR in the accessory portion. Four complete two-component systems presented similarity to known regulators, such as PhoR/PhoP, VicK/VicR, AgrC/AgrA, and CiaH/CiaR.

### Antibiotic Resistance

The search for antibiotic resistance genes with RGI tool did not return any hit marked as “Perfect” or “Strict.” Many lactic acid bacteria possess intrinsic resistance to vancomycin due to D-alanine–D-alanine ligase encoded by the gene *ddl*. This resistance is correlated to the presence of a conserved phenylalanine residue (F) in the active site of the enzyme, whereas the sensitive strains possess a tyrosine residue (Y) in this position (Campedelli et al., 2019). The multiple sequence alignment of the *ddl* enzyme highlighted that all the 17 strains of *L. carnosum* possess the F-type enzyme, resulting in intrinsic vancomycin resistance.

### Bacteriophages and Phage Defense

The genome sequences were investigated for prophages using PHASTER. For each strain, the tool identified 1–4 prophage gene clusters. Several intact prophages and a few incomplete ones were detected (Supplementary Table 2). Complete sequences of putatively active prophages were found in 9 out of 17 genomes (WC0319, WC0320, WC0321, WC0322, WC0323, WC0324, WC0325, WC0328, and MFPC16A2803). In all the strains without active prophages, sequences of prophages likely inactive were identified. In three strains, WC0321, WC0328, and CBA3620, questionable regions have also been found.

A type II CRISPR-Cas locus was found in the chromosomes of *L. carnosum* WC0319 and WC0324. The CRISPR array of WC0319 and WC0324 contained 16 and 11 spacers, respectively, the 11 of WC0324 identified as a first string of WC319 array, followed by other five unshared spacers. This arrangement suggested an iterative acquisition of the spacers and a common origin of the two strains, in agreement with the negligible phylogenetic distance, with the close position in the PCoA plot, with the hold of single similar plasmids (pELI19 and pELI24).

### Bacteriocin Production

Screening of the entire genomes of *L. carnosum* using the bacteriocin database BAGEL 4 revealed the presence of five types of putative bacteriocin encoding loci (Table 3). Plasmids of *L. carnosum* WC0321, WC0323, and WC0329 harbored the genes for the synthesis of leucocin B. The genome of *L. carnosum* WC0321 and WC0323 encompassed also the gene encoding the transporter LanT, missing in WC0329. Most of the strains encoded a chromosomal lactococcin-like protein with double glycine leader peptide. No further information on the type of lactococcin was yielded by the prediction tool. Genes encoding mesentericin B105, mesentericin Y105, and/or a mesentericin-like protein were found in the genome of *L. carnosum* WC0328 and *L. carnosum* MFPC16A2803.

**TABLE 3:** Putative bacteriocins genes present in the strains.

Strain	Bacteriocin
WC0318	Lactococcin-like bacteriocin
WC0319	–
WC0320	Lactococcin-like bacteriocin
WC0321	Leucocin B (pFRA21) and lactococcin-like bacteriocin
WC0322	Lactococcin-like bacteriocin
WC0323	Leucocin B (pFRA23) and lactococcin-like bacteriocin
WC0324	–
WC0325	Lactococcin-like bacteriocin
WC0326	Lactococcin-like bacteriocin
WC0327	Lactococcin-like bacteriocin
WC0328	Mesentericin B105 and mesentericin Y105
WC0329	Leucocin B (pFRO29) and lactococcin-like bacteriocin
JB16	–
CBA3620	–
MFPC16A2803	Lactococcin-like bacteriocin, mesentericin-like protein, and mesentericin B105
MFPA29A1405	Lactococcin-like bacteriocin
DSM 5576T	–

*Genes are chromosomal unless a plasmid indication is given in brackets. – indicates absence of putative bacteriocins.*

## Discussion

In this study, all the genomes available for *L. carnosum* were compared to decipher their metabolic and functional potential and to determine the ecological role in food matrices (mainly meat) and the impact on food transformations. Most strains (15) were isolated from packed meat products of various origin (cooked ham, sausages, and beef), whereas a minority were isolated from kimchi (Jung et al., 2012; Raimondi et al., 2018, 2019). Genome comparison indicated that the 17 strains of *L. carnosum* are a compact group of bacteria, with values of sequence similarity much higher than the threshold required for species demarcation (ddDH > 90.5%; ANI > 98.9%), albeit their pangenome remained open. Differences within the core genome, revealed by sequence alignment, indicated three clades of strains. The clade encompassing most of the strains, all isolated from meat, was also separated in the PCoA plot computed from the matrix of the presence/absence of genes. The two strains isolated from kimchi were closely related, laying in the same clade and PCoA cluster, together with meat strains. Differences of location in PCoA plot with respect to phylogenetic distances can be attributed to accessory genes, with some contribution of genes encoded by plasmids. For instance, *L. carnosum* WC0328 is in a different clade than *L. carnosum* WC0324 and WC0319, but they all cluster together in the PCoA plot and harbor a plasmid of the pELI series. *L. carnosum* WC326 and WC327, which resulted to be the same strain, were isolated from the same factory, in fresh sausages in diverse lots sampled 2 months apart (Raimondi et al., 2018), suggesting the persistence of this species in the environment of production plants.

*Leuconostoc carnosum* harbored the genes for heterolactic fermentation sugars, yielding L-lactate, ethanol, acetate, and CO_2_ (Posthuma et al., 2002). The availability in meat of a small range of carbohydrates, mainly glucose deriving from glycogen hydrolysis (Pereira and Vicente, 2013), is consistent with the small potential to ferment sugars, restricted to glucose and few others. With a sole exception, carbohydrate metabolic capabilities were shared by all the strains and were not strain-dependent. The genes for ribose assimilation and fermentation were present only in *L. carnosum* WC0322, encoded by plasmid pLQ22. Unlike other lactic acid bacteria, including *Leuconostoc* species, *L. carnosum* seemed unable to uptake and metabolize citrate *via* citrate lyase and oxaloacetate decarboxylase (Bekal et al., 1998; García-Quintáns et al., 2008). However, all the strains may yield flavored four carbon metabolites, such as acetoin and butanediol from pyruvate, even though the role of *L. carnosum* in food ripening and aroma development remains to be clarified.

Meat is a rich substrate mainly composed of proteins, albeit an abundant fraction of amino acids or small peptides is also available (Cobos and Díaz, 2015). Despite the ability to synthetize some amino acids, all the strains seemed unable to grow utilizing ammonium as the sole nitrogen source and must rely on a pool of few pre-formed amino acids that should include at least glutamate, aspartate, serine, and alanine. Since the biosynthetic pathway of other amino acids (e.g., tyrosine, phenylalanine, and lysine) is present but incomplete, it remains to be clarified whether some genes are not correctly annotated, and prediction could make up for the interruption. Adaptation of *L. carnosum* to a nitrogen-rich environment explains the autotrophy not only for several amino acids but also for some ribonucleotide and deoxyribonucleotide and for several vitamins and cofactors. On the other hand, *L. carnosum* clearly took advantage of the 23 peptidase genes identified in the core genome and the other two encoded by accessory genes.

The presence of a choline transporter indicates that *L. carnosum* may use this compound that is abundant in meat to cope with salt and other osmotic stress. However, while in other bacteria (e.g., *Bacillus subtilis*) choline uptake is functional to the biosynthesis of the osmoprotectant betaine (Kappes et al., 1999), any gene involved in choline transformation to betaine is missing.

All the strains herein compared harbored 1–4 plasmids that presented main functions associated to: hydrolysis of proteins; transport and metabolism of amino acids, oligopeptides, and carbohydrates; production of bacteriocin and exopolysaccharide; resistance to bacteriophage, heavy metal, and other stress responses; and DNA restriction–modification systems. A peculiar trait of these replicons was the absence of genetic determinants for antibiotic resistance that were not detected either in the chromosomes. However, the genes *mdlAB* and *lmrCD*, putatively encoding the multidrug efflux systems, were located in the genome. There is no information on whether *mdlAB* confers any antibiotic resistance to bacteria, whereas *lmrCD* confers to *Lactococcus lactis* the resistance to some toxic compounds, including daunomycin (Kobayashi et al., 2001; Lubelski et al., 2006). The lack of genes encoding antibiotic resistance may be due to the specificity of *L. carnosum* for meat matrices that seclude the species in an environment where the selective pressure is limited and the interaction with gut bacteria, the most exposed to the antibiotics utilized in animal production, is low. The safety of *L. carnosum* is a key point, considering the high load of bacteria belonging to this species that are ingested mostly by consuming products, such as MAP sliced meat. Safety of this species was associated to the absence not only of any antibiotic resistance genetic determinants but also of decarboxylase genes for biogenic amine production, assessing the incapability of these strains to produce biogenic amine.

In most of the strains, the potential of bacteriocin synthesis, secretion, and immunity was identified, including the production of leucocin B, a class IIa bacteriocin effective against *L. monocytogenes* (Felix et al., 1994). It is quite interesting the fact that, at the end of the shelf-life, MAP cooked ham generally presented an abundant population of *L. carnosum*, regardless of the sensorial evolution of the product (Raimondi et al., 2019). Indeed, *L. carnosum* dominated both in seriously spoiled products and in those that maintained good sensorial properties. However, studies of biochemistry and physiology of *L. carnosum*, also focused on molecular mechanisms affecting meat spoilage, are still lacking. The utilization of *L. carnosum* strains as bioprotective starters could be encouraged by the ability to produce bacteriocins; thus, a deeper investigation is required to determine their activity spectrum against spoilage and pathogen bacteria. Production and activity of the predicted lactococcin-like proteins need to be analyzed further.

As a whole, this study provided a deep insight into the genomic and metabolic features of this important bacterium ubiquitous in meat products, revealing that it is safe and opening new possibilities of exploitation in the food conservation industry.

## Data Availability Statement

The datasets presented in this study can be found in online repositories. The names of the repository/repositories and accession number(s) can be found in the article/Supplementary Material.

## Author Contributions

SR, JS, and MR conceived and designed the experiments. FC carried out the DNA extraction and the bioinformatics for genome assembly and annotation. MT supervised genome sequencing, assembly, and annotation. FC, GS, and AA performed data analysis. SR, AA, and MR wrote the manuscript with contributions from all other authors. All authors contributed to the article and approved the submitted version.

## Conflict of Interest

The authors declare that the research was conducted in the absence of any commercial or financial relationships that could be construed as a potential conflict of interest.

## Footnotes

1 http://enve-omics.ce.gatech.edu/g-matrix/

2 https://www.dsmz.de/services/online-tools/genome-to-genome-distance-calculator-ggdc

3 http://phaster.ca/

4 https://crisprcas.i2bc.paris-saclay.fr/CrisprCasFinder/Index

5 http://bagel4.molgenrug.nl/index.php

6 https://isfinder.biotoul.fr/

7 https://card.mcmaster.ca/analyze/rgi

8 http://weizhong-lab.ucsd.edu/webMGA/server/

9 https://www.kegg.jp/blastkoala

10 https://www.genome.jp/kegg/tool/map_pathway.html
