# Supplementary material for: Comparative Genomics of Leuconostoc carnosum
Source: Front Microbiol. 2021 Jan 11;11:605127. doi: 10.3389/fmicb.2020.605127 (PMC7829361; doi:10.3389/fmicb.2020.605127)
Supplement: Supplementary file 1 [file Table_1.pdf]

Comparative genomics of *Leuconostoc carnosum*

Francesco Candeliere [a], Stefano Raimondi [a], Gloria Spampinato [a], Moon Yue Feng Tay [c,d], Alberto Amaretti [a,b], Joergen Schlundt [c,d], Maddalena Rossi [a,b, #]

[a] Department of Life Sciences, University of Modena and Reggio Emilia, Modena, Italy

[b] Biogest-Siteia, University of Modena and Reggio Emilia, Modena, Reggio Emilia, Italy

[c] Nanyang Technological University Food Technology Centre (NAFTEC), Singapore

[d] School of Chemical and Biomedical Engineering, Nanyang Technological University, Singapore

# Address correspondence to: maddalena.rossi@unimore.it

Supplementary Material

Table S1 ANI and dDDH values calculated between the 12 strains analyzed in this work and the 5 publicly available. The blank diagonal line marks 100% value.

	WC0318	WC0319	WC0320	WC0321	WC0322	WC0323	WC0324	WC0325	WC0326	WC0327	WC0328	WC0329	JB16	CBA3620	MFPC16A2803	MFPA29A1405	DSM 5576
WC0318		99.0	99.8	99.7	99.6	99.7	99.0	99.7	99.7	99.7	99.1	99.7	99.1	99.1	99.8	99.8	99.6
WC0319	91.7		98.9	99.0	99.0	99.0	100	99.0	98.9	98.9	99.2	99.0	99.2	99.2	99.0	99.0	98.9
WC0320	98.2	90.8		99.7	99.7	99.8	98.9	99.7	99.8	99.8	99.0	99.8	99.1	99.0	99.8	99.8	99.7
WC0321	97.7	91.6	96.8		99.8	100	99.0	99.8	99.8	99.8	99.1	99.9	99.1	99.2	99.9	100	99.8
WC0322	97.5	91.7	95.5	97.7		99.8	99.0	99.8	99.7	99.7	99.0	99.9	99.1	99.1	99.8	99.9	99.8
WC0323	98.3	91.5	97.4	99.6	97.9		99.0	99.8	99.8	99.8	99.1	99.9	99.1	99.2	99.9	100	99.9
WC0324	91.7	99.7	92.2	90.7	91.4	91.4		99.0	98.9	98.9	99.1	99.0	99.2	99.2	99.0	99.0	98.9
WC0325	97.4	92.1	97.1	97.2	96.9	97.6	91.7		99.7	99.7	99.1	99.9	99.2	99.1	99.9	99.9	99.8
WC0326	98.0	91.0	98.0	98.1	98.2	98.2	91.0	97.6		100.0	99.1	99.8	99.1	99.1	99.9	99.8	99.7
WC0327	98.0	91.0	98.0	98.1	98.2	98.2	91.0	97.7	100		99.1	99.8	99.1	99.1	99.9	99.8	99.7
WC0328	92.6	93.4	90.5	91.8	90.6	92.3	92.9	91.3	92.3	92.3		99.1	100	99.8	99.2	99.2	99.1
WC0329	98.3	91.7	98.3	99.6	99.4	99.6	91.8	99.2	98.4	98.4	93.3		99.2	99.2	99.9	100	99.9
JB16	93.2	94.2	92.9	93.4	92.1	93.4	94.2	93.4	92.7	92.7	99.7	93.8		99.9	99.2	99.2	99.1
CBA3620	92.9	93.8	92.2	93.8	92.5	93.8	93.8	93.4	92.7	92.7	98.4	93.8	99.1		99.1	99.2	99.1
MFPC16A2803	98.8	92.1	98.1	99.1	97.4	98.7	91.9	98.9	99.2	99.2	92.6	99.5	93.6	93.4		99.9	99.9
MFPA29A1405	98.5	92.2	98.7	99.7	99.3	99.6	92.2	99.0	98.9	98.9	93.6	100	93.7	93.8	100		99.9
DSM 5576T	96.7	91.3	96.7	98.2	97.2	98.7	91.5	98.6	97.1	97.1	92.1	99.1	91.9	93.5	99.6	99.4	

ANI

dDDH

Table S2 Prophage sequences predicted in each strain

Strains	Region	Region length [kb]	Completeness	Region position	Specific keywords
WC0318	1	7.7	incomplete	NODE_1:255985-263748	terminase
	2	12.1	incomplete	NODE_1:617214-629331	NA
WC0319	1	56.2	intact	NODE_1:910712-966945	plate,tail,capsid,head,portal,terminase,integrase
WC0320	1	39.3	intact	NODE_1:190577-229913	integrase,head,terminase,capsid,tail,lysin
WC0321	1	22.7	questionable	NODE_1:545803-568571	integrase,terminase,portal,head,capsid
	2	27.5	intact	NODE_2:159280-186818	lysin,tail,head,protease,portal,terminase
WC0322	1	20.5	incomplete	NODE_2:10793-31363	terminase,portal,protease,integrase
	2	30.4	incomplete	NODE_2:275889-306303	integrase
	3	37.5	intact	NODE_4:159079-196638	tail,capsid,terminase,integrase
WC0323	1	16.1	incomplete	NODE_2:285942-302126	integrase
	2	38	intact	NODE_4:159025-197044	lysin,tail,capsid,terminase,integrase
WC0324	1	56.2	intact	NODE_1:910707-966986	plate,tail,capsid,head,portal,terminase,integrase
	2	52.5	intact	NODE_2:142206-194757	lysin,plate,tail,head,protease,portal,terminase,integrase
	3	20.8	incomplete	NODE_5:227-21106	recombinase,transposase,integrase
WC0325	1	18.4	incomplete	NODE_5:123842-142309	lysis
	2	28.5	incomplete	NODE_2:268998-29758	integrase
	3	21.5	intact	NODE_10:1048-22588	terminase,portal,protease,head,tail,capsid
	4	21.7	intact	NODE_11:223-21970	terminase,capsid,tail
WC0326	1	6.6	incomplete	NODE_6:3622-10245	transposase,recombinase
WC0327	1	6.6	incomplete	NODE_6:3622-10245	transposase,recombinase
WC0328	1	43.6	intact	NODE_4:154101-197752	tail,head,protease,portal,terminase,integrase
	2	18	questionable	NODE_3:96311-114367	integrase,capsid,protease,portal,terminase
	3	11.3	incomplete	NODE_7:42902-54207	recombinase,transposase,integrase
WC0329	1	16.1	incomplete	NODE_2:276411-292595	integrase
JB16	1	11.3	incomplete	plasmid pKLC2:6267-17584	recombinase,transposase,integrase
CBA3620	1	19.6	questionable	NZ_CP042374.1:1055468-1075152	terminase,portal,protease,capsid,integrase
	2	9.5	incomplete	plasmid unnamed1:3-9544	transposase
	3	4.6	incomplete	plasmid unnamed2:39413-44024	transposase,plate
MFPC16A2803	1	41.3	intact	NZ_ONZM01000028.1:135658-176964	integrase, terminase,head,portal,protease,capsid,tail
	2	30.4	incomplete	NZ_ONZM01000029.1:242566-272980	integrase
MFPA29A1405	1	16.1	incomplete	NZ_OOIO01000002.1:236592-252776	integrase
DSM 5576T	1	30.4	incomplete	NZ_JACHGL010000002.1:277838-308252	integrase

Gene	Annotation	WC0318	WC0319	WC0320	WC0321	WC0322	WC0323	WC0324	WC0325	WC0326	WC0327	WC0328	WC0329	JB16	CBA3620	MFPC16A2803	MFPA29A1405	DSM 5576T
pepS	Aminopeptidase [Leuconostoc carnosum]	■	■	■	■	■	■	■	■	■	■	■	■	■	■	■	■	■
pepC	Aminopeptidase C [Leuconostoc carnosum]	■	■	■	■	■	■	■	■	■	■	■	■	■	■	■	■	■
C270_RS06005	Aminopeptidase N [Leuconostoc carnosum]	■	■	■	■	■	■	■	■	■	■	■	■	■	■	■	■	■
C270_RS05935	Aminopeptidase P family protein [Leuconostoc carnosum]	■	■	■	■	■	■	■	■	■	■	■	■	■	■	■	■	■
C270_RS03565	Aminopeptidase P family protein [Leuconostoc carnosum]	■	■	■	■	■	■	■	■	■	■	■	■	■	■	■	■	■
C270_RS03665	Carboxypeptidase [Leuconostoc carnosum]	■	■	■	■	■	■	■	■	■	■	■	■	■	■	■	■	■
C270_RS04865	Carboxypeptidase M32 [Leuconostoc carnosum]	■	■	■	■	■	■	■	■	■	■	■	■	■	■	■	■	■
C270_RS06920	D-alanyl-D-alanine carboxypeptidase [Leuconostoc carnosum]	■	■	■	■	■	■	■	■	■	■	■	■	■	■	■	■	■
pepV	Dipeptidase PepV [Leuconostoc carnosum]	■	■	■	■	■	■	■	■	■	■	■	■	■	■	■	■	■
pepO	Endopeptidase [Leuconostoc carnosum]	■	■	■	■	■	■	■	■	■	■	■	■	■	■	■	■	■
pepA	Glutamyl aminopeptidase [Leuconostoc carnosum]	■	■	■	■	■	■	■	■	■	■	■	■	■	■	■	■	■
C270_RS04365	LD-carboxypeptidase [Leuconostoc carnosum]	■	■	■	■	■	■	■	■	■	■	■	■	■	■	■	■	■
pepN	oligoendopeptidase [Leuconostoc carnosum]	■	■	■	■	■	■	■	■	■	■	■	■	■	■	■	■	■
pepF	oligoendopeptidase F [Leuconostoc carnosum]	■	■	■	■	■	■	■	■	■	■	■	■	■	■	■	■	■
C270_RS06825	peptidase [Leuconostoc carnosum]	■	■	■	■	■	■	■	■	■	■	■	■	■	■	■	■	■
C270_RS04305	peptidase E [Leuconostoc carnosum]	■	■	■	■	■	■	■	■	■	■	■	■	■	■	■	■	■
C270_RS01410	peptidase M15 [Leuconostoc carnosum]	■	■	■	■	■	■	■	■	■	■	■	■	■	■	■	■	■
C270_RS01985	peptidase M20 [Leuconostoc carnosum]	■	■	■	■	■	■	■	■	■	■	■	■	■	■	■	■	■
C270_RS07440	peptidase M20 [Leuconostoc carnosum]	■	■	■	■	■	■	■	■	■	■	■	■	■	■	■	■	■
pepT	peptidase T [Leuconostoc carnosum]	■	■	■	■	■	■	■	■	■	■	■	■	■	■	■	■	■
C270_RS00920	prepilin peptidase [Leuconostoc carnosum]	■	■	■	■	■	■	■	■	■	■	■	■	■	■	■	■	■
map	type I methionyl aminopeptidase [Leuconostoc carnosum]	■	■	■	■	■	■	■	■	■	■	■	■	■	■	■	■	■
pepX	Xaa-Pro dipeptidyl-peptidase [Leuconostoc carnosum]	■	■	■	■	■	■	■	■	■	■	■	■	■	■	■	■	■
C270_RS01760	C1 family peptidase [Leuconostoc carnosum]											■		■	■	■	■	■
pcp	Pyrrolidone-carboxylate peptidase								■									

FIG S1 Predicted genes encoding peptidases (green) in the genome of *L. carnosum*.
